# NDUFS1 upregulates ENaCα by NAD+ to promote alveolar fluid clearance in acute lung injury

**DOI:** 10.7150/ijms.112248

**Published:** 2025-07-28

**Authors:** Mengmeng Wang, Mengting Chen, Jianping Zhu, Yu Zhang, Jian Lu, Zhiying Yue, Zhengfeng Yang, Ruilan Wang

**Affiliations:** 1 Department of Critical Care Medicine, Shanghai General Hospital, Shanghai Jiao Tong University School of Medicine, Shanghai 201620, China.; 2 Precision Research Center for Refractory Diseases, Shanghai Jiao Tong University Pioneer Research Institute for Molecular and Cell Therapies, Shanghai General Hospital, Shanghai Jiao Tong University School of Medicine, Shanghai, 201620, China; State Key Laboratory of Innovative Immunotherapy, School of Pharmaceutical Sciences, Shanghai Jiao Tong University, Shanghai, 200240, China.; 3 Department of Critical Care Medicine, Shanghai United Family Hospital, Shanghai, 200335, China.

**Keywords:** acute lung injury, NDUFS1, mitochondrial dysfunction, NAD+, ENaCα

## Abstract

Alveolar edema and following respiratory distress results in the aggravation of epithelial damage and the progression of acute lung injury (ALI), however, with unclear molecular mechanism remained to be elucidated. Through proteomic screening and scRNA-seq mining analysis, we detected the decline expression of NDUFS1 in epithelial cells in lungs from paraquat/LPS-induced ALI models. NDUFS1 deficiency in alveolar epithelial cells reduced ENaCα expression, which impaired alveolar fluid clearance (AFC) and led to alveolar edema. Mechanistically, NDUSF1 deficiency in alveolar epithelial cells leads to mitochondrial dysfunction such as reduced complex I activity, impaired NAD+ production and increased ROS, these contributed to the decline of ENaCα. Supplementing NAD+ via Olaparib treatment alleviated the reduction of ENaCα abundance raised by NDUFS1 deficiency, improved AFC, and suppressed the progression of ALI. In summary, our study suggests that NDUFS1 promotes AFC by regulating ENaCα via NAD+ in pulmonary epithelial cells during ALI.

## Introduction

Acute lung injury (ALI) presents diffuse alveolar damage caused by multiple types of insults, including toxins and microbes' infection 1. The pathological changes of ALI mainly include damage to the integrity of alveolar epithelial cells, gas exchange disorders, alveolar edema, and the formation of transparent membranes 2. The progressive ALI can be attributed to moderate or severe acute respiratory distress syndrome (ARDS) 3. Although supportive therapies, including mechanical ventilation, pharmacological intervention, and prone positioning ventilation, can improve the symptoms of ARDS patients, these strategies cannot effectively decrease the mortality rate of ALI/ARDS 4, 5. Therefore, it is still required to understand the precise pathogenesis of ALI to develop improved effective strategies.

Epithelial sodium channel (ENaC) is a structure composed of α, β, and γ subunits and α subunit is essential for the functional ENaC channels 6. Under physiological conditions, the ENaC on the apical membrane of alveolar epithelial cells (AECs) transports sodium ions from the alveolar cavity into the cells, then the Na-K-ATPase on the basement membrane side of AECs transfers them into the lung interstitium 7, which maintains sodium concentration gradient in AECs and thus provide the force to drive fluid from the alveolar space into the pulmonary interstitium. This process is called alveolar fluid clearance (AFC), which keeps the alveolar cavity dry and facilitates air exchange. However, in the early stage of ALI with ENaC deficiency, excess fluid was accumulated in the alveolar space that impairs air exchange and aggravates hypoxia, leading to a further damage to the alveolar epithelium and forming a vicious cycle that promotion of ALI progression 8. Indeed, most of ARDS patients present severe disorders in alveolar fluid clearance, leading to acute respiratory failure together with a high mortality rate 9. Clearing excess fluid from the alveolar cavity and promoting air exchange thus would be the watershed to determine the prognosis of ARDS patients 10. Consistently, several studies show that reducing alveolar edema in the initial stage of ALI alleviates pulmonary inflammation and subsequent injury 11, 12, including indirect pharmacotherapies for AFC via reducing systemic inflammation, however, little strategies developed to directly target AFC via ENaC 7. A combination of anti-inflammatory and promoting alveolar fluid clearance would expect to disturb the vicious progression of ALI.

Multiple factors have been reported to mediate the expression and activity of ENaC 13, including aldosterone, Angiotensin II, and phospholipids, etc. Though each agent induces the expression of ENaC via different signaling cascades, these stimulators share a common route to mediate energy metabolism and the production of reactive oxidative species, which has been well recognized to be dependent on mitochondrial activity 14, 15. The importance of mitochondria in modulation of AFC during ALI has not been well characterized. However, it has been reported that mitochondria play a crucial role in maintaining lung health and modulation of respiratory diseases 16, making it reasonable to assume a potential regulation of ENaC activation and following AFC by mitochondria. Mitochondrial activity can be contributed by the activation of respiratory chain complexes, from which complex I is a rate-limiting step in the overall electron transfer and essential for mitochondrial oxidative respiration and following energy production 17. NDUFS1 is the largest core subunit of mitochondrial respiratory chain complex I and regulates the generation of NAD+ 18. NDUFS1 has been reported to modulate tissue or cell injury in certain scenarios, including cardiac dysfunction and myocardial fibrosis after myocardial infarction 19, and the enhancement of radiation sensitivity to cancer cells for suppressing tumor progression 20, 21, etc. Here, we further identified the importance of NDUFS1 in regulation of paraquat (PQ)/lipopolysaccharide (LPS)-induced acute lung injury via modulation of ENaCα production, expanding the understanding of mitochondrial activity in AFC and acute lung injury.

## Materials and Methods

### Animals and treatments

For PQ-induced ALI model, 8-12 weeks old C57BL/6 male mice were intraperitoneally injected with 40 mg/kg PQ one time 22 and sacrificed at day 3. For LPS-induced ALI model, 6-8 weeks old male mice were treated with 5mg/kg LPS via the non-surgical intratracheal instillation. For additional NAD supplementation, 5mg/kg Olaparib were intraperitoneally injected in the ALI mice model. For inhibiting NDUFS1 in mice lung tissues, 3mg/kg Rotenone were intraperitoneally injected in mice. PQ and LPS were purchased from Sigma-Aldrich (St. Louis, MO, USA). Rotenone and Olaparib were bought from Med Chem Express (NJ, USA). All of the mice were fed at the Shanghai General Hospital Laboratory Animal Center. The mice were housed with a 12h day/night cycles and free to water and food for 72 hours before this study. The animal experiments have been approved by the Animal Ethics Committee of the Shanghai General Hospital.

### Cell culture

The A549 human epithelial cell line was bought from Cell Bank of the Chinese Academy of Sciences (Shanghai, China), and cultured in DMEM medium (Gibco, USA) containing 10% fetal bovine serum (Gibco, USA) at 37 °C incubator with 5% carbon dioxide. The mediums of cells were changed every 2-3 days. To mimic PQ-induced epithelial cell damage, A549 cells were treated with PQ at a final concentration of 400 µM for 24 hours and then we collected cells for subsequent experiments. To mimic LPS-induced epithelial cell damage, we used LPS at a final concentration of 10 µg/ml to stimulate A549 cells for 12 hours.

### Western blotting

Cells and mice tissues were collected and the proteins were extracted with RIPA lysis buffer (Beyotime, P0013C) on the ice. Then we use the BCA protein assay kit (Thermo Fisher Scientific, 23225) to detect protein concentration. Equal amounts of protein (20-40μg) were separated by 7.5% or 10% SDS-PAGE gels (Epizyme Biotech, PG111; Epizyme Biotech, PG112) and transferred to nitrocellulose filter membranes (Pall, 66485). The membranes were blocked with 5% skimmed milk at room temperature (RT) for 1-2 hours. Then we incubated these membranes with primary antibodies at 40 °C overnight, including anti-E-cadherin (Proteintech, 60335-1-IG-50UL,1:2000), anti-Vimentin (Cell Signaling Technology, 5741S, 1:1000), anti-ENaCα (Proteintech, 28707-1AP, 1:1000), anti-SFTPC (Abcam, ab90716, 1:1000), anti-NDUFS1antibody (Abcam, ab169540, 1:2000), anti-GAPDH (Affinity, AF7021, 1:3000). We washed these membranes with Tris-buffered saline with Tween 20 (TBST) for about 6 minutes for 6 times in total, after which horseradish peroxidase-labeled secondary antibody (Beyotime A0216, A0208;1:2000) was incubated with membranes at RT for 1 hour. The membranes were washed for about 6 minutes for 6 times with TBST. Target proteins were detected by chemiluminescence detection system (Thermo Fisher Scientific, USA).

### Quantitative reverse-transcription (qRT)-PCR assays

Cells and mice tissues were harvested and total RNA were extracted with RNA-easy^TM^ Isolation Reagent (Vazyme, Nanjing, China). The HiScript III RT SuperMix kit (Vazyme Biotech Co, R323) was used to reverse transcribe the total RNA to cDNA. Cham Q SYBR Color qPCR Master Mix (Vazyme Biotech Co, Q431-02) was used to perform qRT-PCR to detect the relative mRNA expression of *b-actin*,* Ndufs1*, *Ndufs2*, *Ndufs3*, *Ndufs7*, *Ndufs8*, *Ndufv1*,* Ndufv2* and *ENaCα*. We used *β-actin* to normalize the data and calculated using 2^ [- (Ct target gene-Ct β-actin)]^. The sequences of the PCR primers are available in Table S1.

### Immunofluorescence staining

The lung tissues from LPS-induced ALI mice model were fixed within 4% paraformaldehyde, embedded in paraffin, and sliced into 4-μm-thick sections. The slices were then placed in an incubator at 60℃ for 1 hour, followed by deparaffinization, rehydration, and antigen retrieval by microwave oven (high fire 6 minutes and low fire 10 minutes) in EDTA antigen retrieval solution (BBI, E673003-0250). Next, we used 5% bovine serum albumin to block the slices at RT for 1 hour, then these slices were incubated overnight at 4 °C with primary antibodies, followed by the incubation of indicated secondary antibodies (1:2000) for 1 hour at RT. The primary antibodies utilized included rat anti-SFTPC (Abcam, ab90716, 1:1000) and mouse anti-NDUFS1 (Proteintech, 68253-1-IG, 1:600). The secondary antibodies included Goat anti Mouse IgG (H+) Alexa Fluor 488 (Invitrogen, A32723) and Goat anti Rabbit IgG (H+) Alexa Fluor 594 (Invitrogen, A32740). After washing 5 minutes with PBS for 3 times, these slides were mounted with DAPI Fluoromount-GTM (Yeasen, China). The samples were observed and images were captured with laser scanning confocal microscopy (Leica TCS SP8, Leica).

### Lentivirus generation and cell infection

#### Plasmids

Human NDUFS1 cloned in pLV-mPuro-C-GFPSpark was purchased from Sino Biological.

#### Lentivirus generation and cell infection

HEK293T cells were seeded in 6 cm dishes, then we added a mixture of 2 µg shRNA targeting NDUFS1 (5'- GCAGTAGAGGAACCATCCATA-3'; 5'-TAACCTTTGTGACGAACATAA-3'), 2 µg packaging assistance plasmid (psPAX2), 2 µg envelope plasmid (pMD2.G) and Polyjet transfection reagent in to cells. After 6-8h, the medium was removed and cells were cultured with fresh medium for 24 hours. Then lentivirus supernatant was collected to infect A549 cells. A549 cells were seeded in 6-well plates, cultured with 1.5 ml lentivirus supernatant and 1 ml DMEM containing 10% FBS for 24h for infection, after which the infected cells were selected with 7 µg/ml puromycin for 48-72h. The stable transient cell lines were maintained by adding 5 µg/ml puromycin in culture medium. Similar procedures were performed to generate NDUFS1 overexpression A549 cells.

### Histological staining and assessment of lung injury

The left lung tissues were fixed at 4% paraformaldehyde after mice sacrificed, embedded in paraffin and sliced 4-6 µm for histological staining. These sections were dewaxed and rehydrated before staining. We followed standard procedures to make Hematoxylin and eosin (H&E) staining with H&E staining kits (Servicebio, China). Subsequently, the sections were imaged in a 40x optical microscope. Lung injury was assessed with semiquantitative lung injury scoring system 23.

### Intracellular ROS generation

Infected A549 cells were plated in 24-well plates with a density of 3 × 10^4^ cells per well and cultured overnight. The attached cells were added with 500 µl fresh DMEM culture medium containing 10 µM DCFH-DA (Beyotime, S0033S) and incubated at 37 °C for 30 minutes. Immediately after removing DCFH-DA working solution, we used PBS to wash cells for 3 times and imaged them with a fluorescence microscope (Leica).

### Mitochondrial complex I activity assay

We detected the activity of mitochondrial complex I in NDUFS1 deficient epithelial cells with a complex I enzymatic activity assay kit (Abbkine, Wuhan, China). The protein content was determined by a BCA protein assay kit (Thermo Fisher Scientific, 23225).

### NAD+/NADH assay

We measured the NAD+/NADH levels in cells with NAD+/NADH Assay Kit (Beyotime, S0175) following the manufacturer's instructions. A549 cells were plated in 6-well plates and added with 200 µL extracting solution. We used a multifunctional microplate reader to measure and record 450nm absorbance. Then we detected protein content using a BCA protein assay kit (Thermo Fisher Scientific, 23225).

### SRB assay

A549 cells infected with shRNA (NC) were plated in 96-well plates with a density of 0.5 × 10^4^ cells per well. Then 0, 3.12, 6.25, 12.5, 25, or 50 µM Olaparib were added to stimulate the cells for 24h. The cells were fixed with 10% TCA at 4 ºC for 1h and washed with flowing water 5 times. Next, the cells were stained with 0.4% SRB for 10 minutes at room temperature. Immediately, we used 1% acetic acid to wash cells for 5 times and dried out 96 well plates. Then we dissolved the SRB in cells with 50 µl of 10 mM Tris base buffer per well. Absorbance at 515nm was measured using a multifunctional microplate reader.

### Wet/dry ratio (W/D) and total lung water content of lung tissues

To assess the severity of pulmonary edema, we weighed a part of lung tissue recorded as the wet weight. Then we transferred the lung tissues to an oven at 65 ºC until the lung weight is no longer changing, we recorded the weight as the dry weight. The ratio of wet weight to dry weight was calculated as W/D ratios. Total lung water content is calculated using the following formula: (wet weight - dry weight)/dry weight.

### Single-cell data processing

The scRNA-seq dataset for LPS-induced acute lung injury was obtained from the GEO database (GSE276682).

### Statistics

Data were stated as the mean ± standard deviation. We used student's t-test to analyze the differences between two groups. We used one-way analysis of variance (ANOVA) to analyze the differences between multiple groups. P < 0.05 was considered as a significant difference.

## Results

### NDUFS1 expression is declined in alveolar epithelial cells during acute lung injury progression by proteomics combined with single-cell RNA sequencing analysis

**Figure 1:**
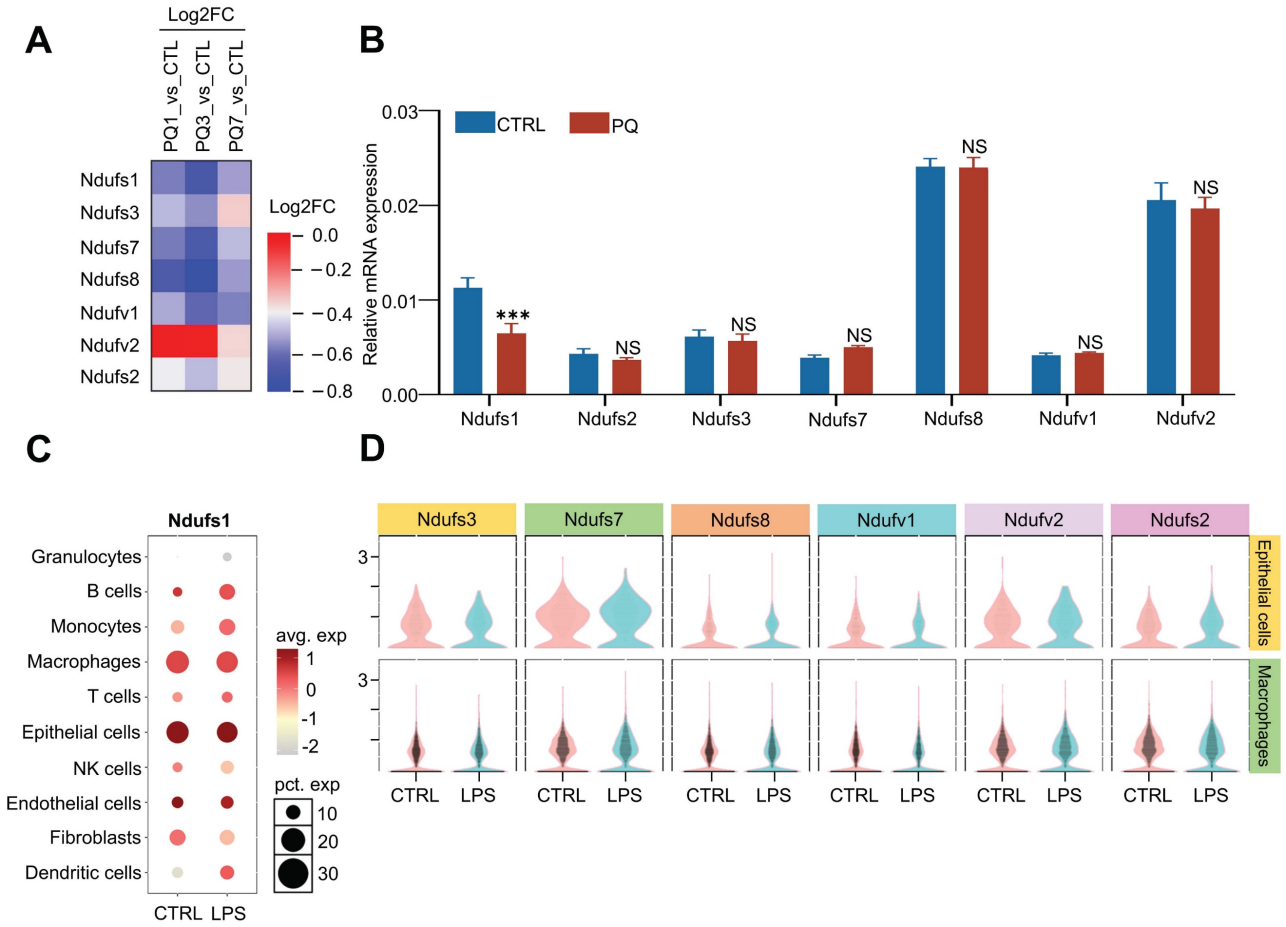
** Proteomics combined with single-cell RNA sequencing analysis identifies that NDUFS1 declined in alveolar epithelial cells during acute lung injury progression. (A)** Proteomic analysis of the expression of seven core subunits of complex I in lung tissues from PQ-induced ALI mice model. Mice were treated with PQ for 1 day (ALI 1), 3days (ALI 3) and & 7days (ALI 7), respectively. n=3 mice per group. PQ, paraquat. **(B)** The mRNA expression levels of Ndufs1, Nudfs2, Ndufs3, Ndufs7, Ndufs8, Ndufv1 and Ndufv2 in A549 cells stimulated with 400 µM PQ for 24 h. **(C)** Bubble plots indicating the presence of Ndufs1 in 10 cell types from lung tissues of CTRL and LPS-induced ALI mice model derived from the single cell-sequencing data of GSE276682. LPS, lipopolysaccharide. **(D)** Violin plots show the expression of other core submits in epithelial cells and macrophages of CTRL and LPS-induced ALI mice model derived from the single cell-sequencing data of GSE276682. Data were presented as mean ± SD. **p < 0.01, ***p < 0.001. NS, not statistically significant.

A proteomics screening found that the abundance of multiple subunits in the mitochondrial membrane respiratory chains was dynamically regulated during PQ- induced acute lung injury (ALI) progression (Fig.1A). Specifically, the protein abundance of NDUFS1, NDUFS3, NDUFS7, NDUFS8, and NDUFV1 were significantly reduced at day 3 and recovered at day 7 after PQ treatment. Considering day 3 after PQ treatment present severe acute lung injury symptom while mice survived after 7 days with PQ treatment were gradually recovered after lung injury, mitochondrial respiratory chains were probably activated in response to ALI progression. We next monitored the mRNA expression of these seven core subunits in A549 cells, the pulmonary epithelial cells, stimulated with or without PQ (Fig.1B). Interestingly, only the mRNA expression of NDUFS1 was specifically and significantly decreased after PQ stimulation. Given that NDUFS1 is the largest subunit of complex I and it plays an important role in complex I function 18, we assumed that NDUFS1-mediated mitochondrial respiratory chain complex I would be most important for mitochondria activity in pulmonary epithelial cells in response to ALI. To further reveal the specificity and potential importance of NDUFS1 in pulmonary epithelial cells, we performed single-cell data mining analysis on LPS-induced ALI mice (GSE276682) and identified that NDUFS1 was mainly enriched in epithelial cells and macrophages, with the expression of NDUFS1 decreased in epithelial cells with LPS stimulation (Fig.1C). Next, we analyzed the expression of other six core subunits in epithelial cells and macrophages on LPS-induced ALI mice (GSE276682) and discovered that they were enriched in epithelial cells (Fig.1D). However, compared with the control group, their expression changes in the LPS group were slight. Even the changes of some submits were opposite to those in proteomics and *in vitro* experiments before. These data indicated that NDUFS1 expression decreased in ALI and the change is relatively stable.

### The NDUFS1 in pulmonary epithelial cells is further confirmed to decrease in PQ/LPS-induced acute lung injury models

**Figure 2:**
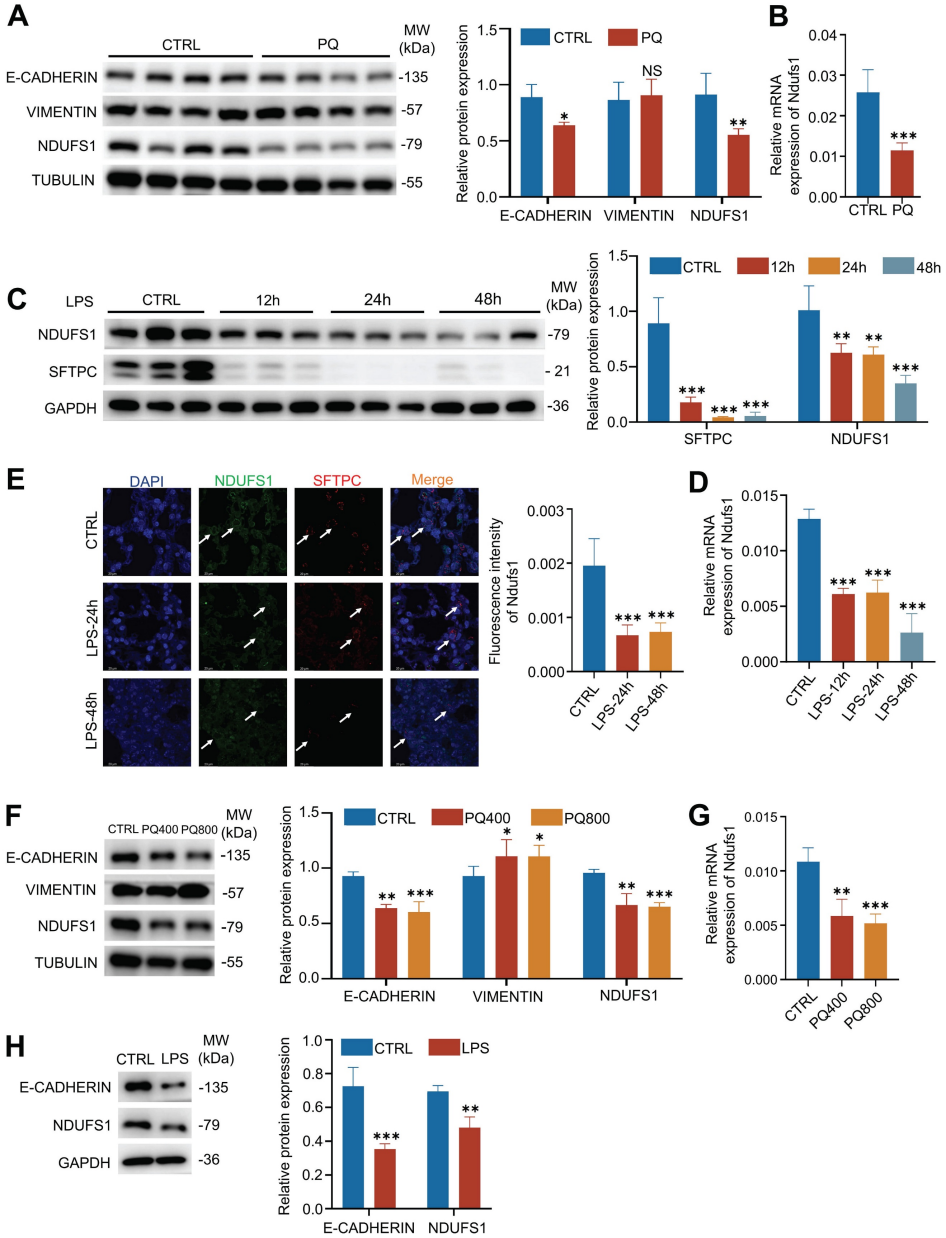
** The expression of NDUFS1 is confirmed to decrease during PQ/LPS-induced acute lung injury. (A-B)** Western blot (A) and QPCR (B) analysis of NDUFS1 expression in lung tissues from PQ-induced ALI mice model. Mice were treated with PQ for 3 days (ALI 3). n=4 mice per group. **(C-D)** Western blot (C) and QPCR (D) analysis of NDUFS1 expression in lung tissues from LPS-induced ALI mice model. Mice were treated with LPS for 12h, 24h and 48h. n=3 mice per group. **(E)** Immunofluorescence analysis of the colocalization signal between SFTPC and NDUFS1 in lung tissues from LPS-induced ALI mice model. Mice were treated with LPS for 24h and 48h. n=3 mice per group. Scale bars: 20 µm. **(F-G)** Western blot (F) and QPCR (G) analysis of E-cadherin, Vimentin or NDUFS1 abundance, as indicated, in A549 cells treated with or without 400 or 800 µM PQ for 24 h. **(H)** Western blot analysis of E-cadherin and Ndufs1 abundance in A549 cells treated with or without 10 µg/ml LPS for 12 h. Data were presented as mean ± SD. *p < 0.05, **p < 0.01, ***p < 0.001. NS, not statistically significant. Unless indicated, data were the representative of two independent experiments.

Next, we used PQ- or LPS-induced ALI models to verify the expression of NDUFS1 during lung injury. Firstly, we established the PQ-induced ALI mice model, indicated by the abundance of E-cadherin. Consistent with the proteomics results, the protein expression of NDUFS1 after lung injury in PQ-treated lung tissues was largely decreased compared with the control group (Fig.2A). Also, the mRNA expression level of NDUFS1 in lung tissues treated with PQ is significantly lower than that in the control group (Fig.2B). Similar results were observed in LPS-induced ALI mice model. Specifically, both protein and mRNA levels of NDUFS1 were decreased in the lung tissues with LPS treatment (Fig.2C-D). Of note, pulmonary surfactant-associated protein C (SFTPC), a surface marker of alveolar type II epithelial cells, was decreased after LPS stimulation, indicating the damage of alveolar epithelial cells. To further confirm that the expression of NDUFS1 was specifically decreased in alveolar type II epithelial cells in ALI, we performed immunofluorescence analysis with observation of the co-staining signal of NDUFS1 and SFTPC. The SFTPC signal is much weaker in the LPS group compared to the control group, indicating that alveolar type II epithelial cells were damaged with LPS stimulation. Importantly, the expression of NDUFS1 in SFTPC+ alveolar type II epithelial cells was decreased in the LPS treated group (Fig.2E), with other types of cells little affected, indicating the specific role of NDUFS1 in alveolar type II cells in modulation of ALI. A further *in vitro* analysis confirmed the potential importance of NDUFS1 in pulmonary epithelial cells. Particularly, both PQ and LPS stimulation reduced the abundance of NDUFS1 in A549 cells, the pulmonary epithelial cells (Fig.2F-H). Collectively, our data suggested that NDUFS1 was specifically decreased in pulmonary epithelial cells during PQ/LPS-induced ALI.

### NDUFS1 deficiency in alveolar epithelial cells leads to alveolar fluid clearance dysfunction by reducing ENaCα

**Figure 3:**
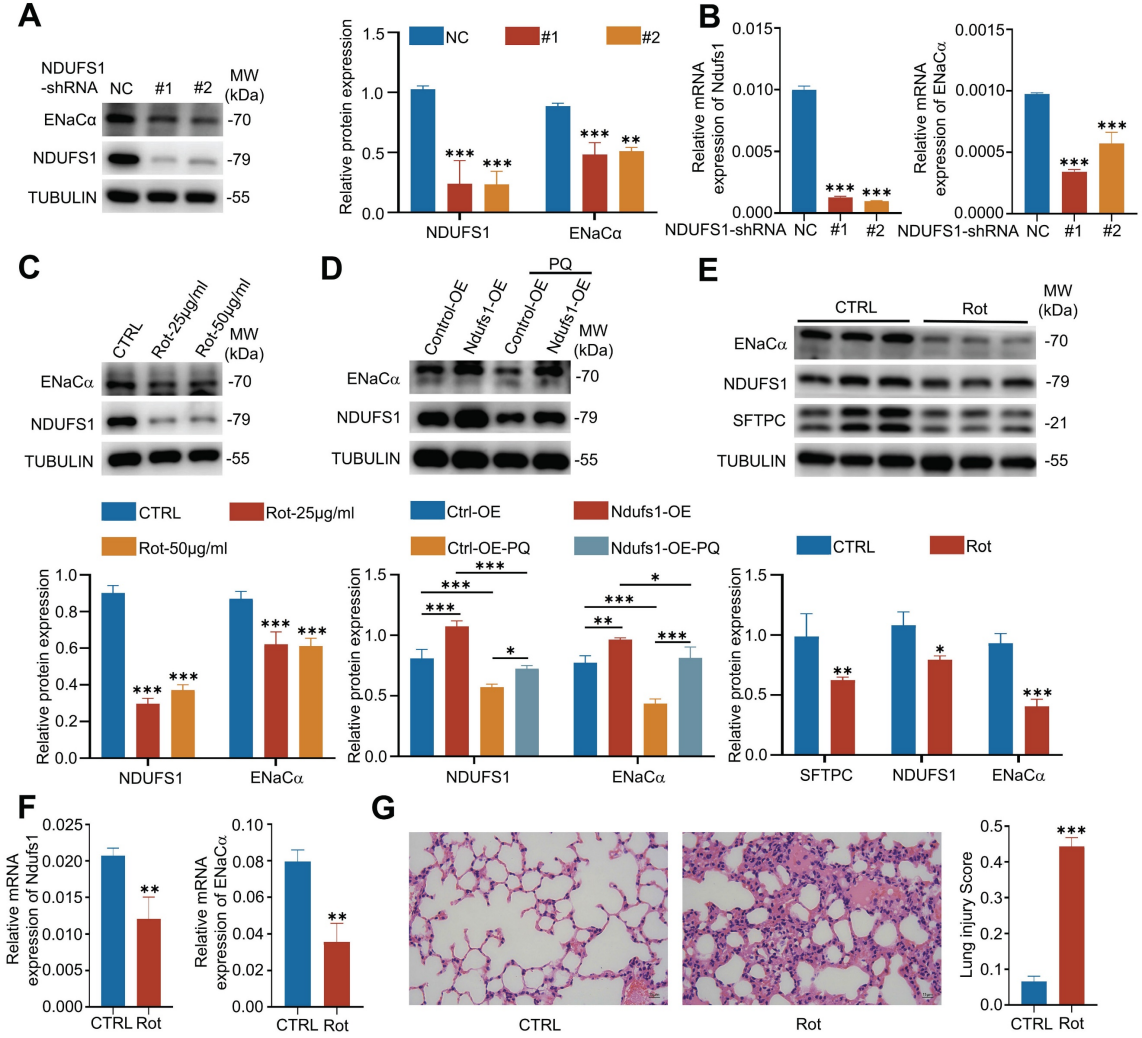
** NDUFS1 mediates the expression of ENaCα* in vitro* and *in vivo.* (A-B)** Western blot (A) and QPCR (B) analysis of NDUFS1 and ENaCα abundance in scrambled shRNA (NC) or NDUFS1 shRNA infected A549 cells. **(C)** Western blot analysis of NDUFS1 and ENaCα abundance in A549 cells treated with or without rotenone with indicated concentrations for 24 h. **(D)** Western blot analysis of NDUFS1 and ENaCα abundance in NC or NDUFS1 overexpression A549 cells treated with or without 400 µM PQ for 24 h. **(E-F)** Western blot (E) and QPCR (F) analysis of NDUFS1 and ENaCα abundance in lung tissues from control (CTRL) and rotenone treated mice. Mice were treated with rotenone for 24h. n=3 mice per group. **(G)** Hematoxylin and eosin staining of lung tissues from control (CTRL) and rotenone treated mice. And the lung injury score of the tissues. Mice were treated with rotenone for 24h. n=3 mice per group. Scale bars: 15 µm. Data were presented as mean ± SD. *p < 0.05, **p < 0.01, ***p < 0.001. Unless indicated, data were the representative of two independent experiments.

The specific reduction of NDUFS1 in pulmonary epithelial cells during ALI indicated the potential role of NDUSF1 in modulation of epithelial cells and ALI progression. Currently, most of ALI/ARDS patients present disorders in alveolar fluid clearance (AFC) and lack effective therapeutic, leading to acute respiratory failure together with a high mortality rate 7, 9. Exploring the mechanisms of AFC may provide new targets for the treatment of ALI. We wondered whether NDUFS1 is involved in the process of AFC. Since the epithelial sodium channel (ENaC) plays a critical role in AFC and the ENaCα is the most important subunit of ENaC 6, 8, we established the NDUFS1 deficient epithelial cells and detect the expression of ENaCα. Our data found that NDUFS1 deficiency alone was sufficient to reduce ENaCα expression (Fig.3A-B). Furthermore, rotenone, the inhibitor of NDUFS1 24, also largely reduced the expression of ENaCα (Fig.3C).

In contrast, NDUFS1 overexpression in pulmonary epithelial cells promoted the expression of ENaCα in the physiological condition and further rescued the downregulated ENaCα expression by PQ stimulation (Fig.3D). These results together suggested that NDUFS1 was required for maintaining the expression of ENaCα in pulmonary epithelial cells. We then further confirmed the correlated expression alteration of NDUFS1 and ENaCα *in vivo*. We injected rotenone in mice intraperitoneally to suppress the expression of NUDFS1 in lung tissues 25. Our data further suggested that the NDUFS1 deficiency in mice lung tissues reduced the expression of ENaCα (Fig.3E-F). Indeed, HE staining of pathological sections revealed that NDUFS1 deficiency led to severe lung injury accompanied with alveolar flooding in the physiological condition (Fig.3G). These data together suggested that NDUFS1 deficiency led to AFC dysfunction via impairment of ENaCα in pulmonary epithelial cells, and further promoted the pulmonary edema.

### NDUFS1 gene knockdown reduces the expression of ENaCα in alveolar epithelial cells via mitochondrial dysfunction

**Figure 4:**
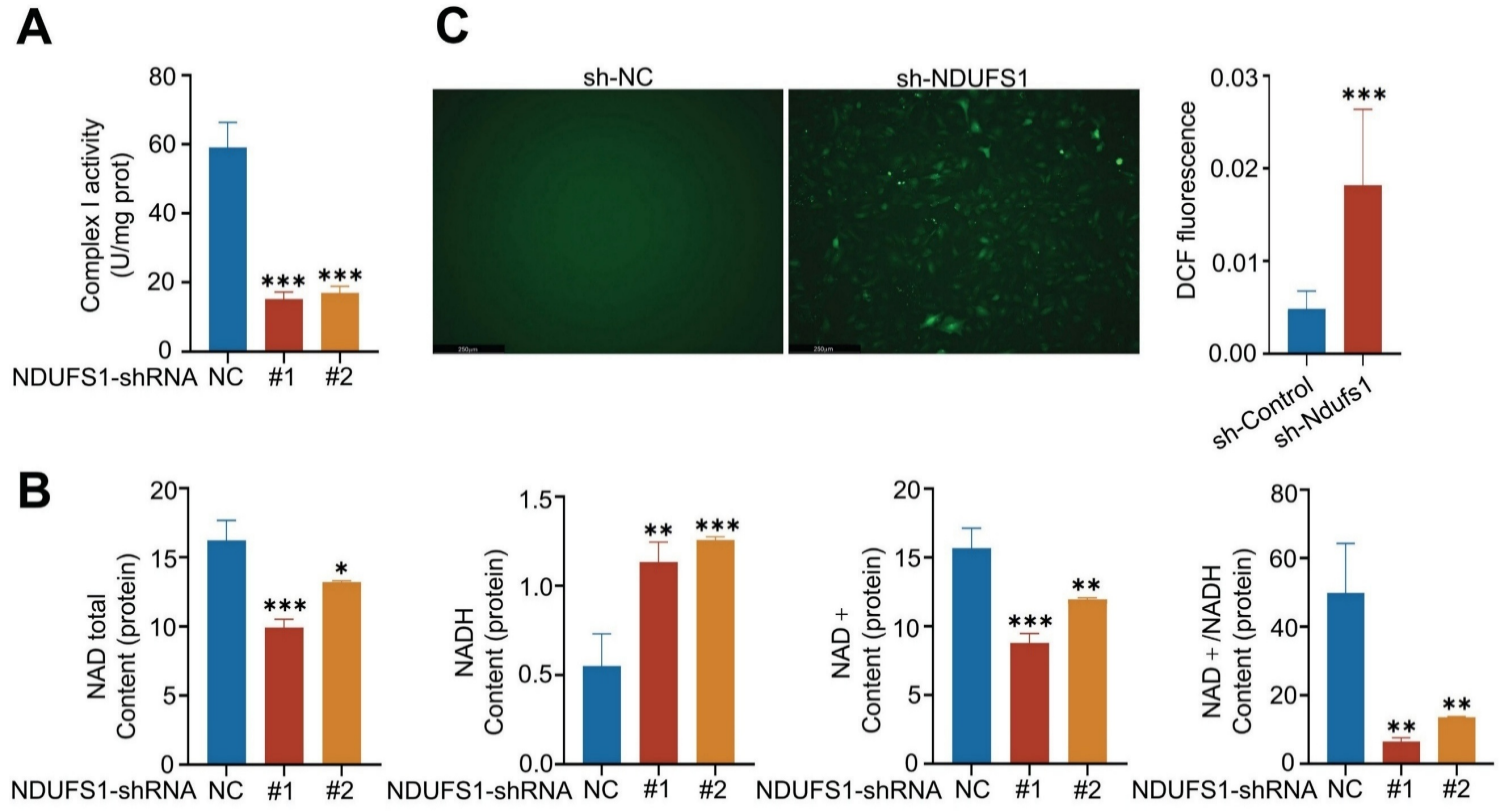
** NDUFS1 deficiency in pulmonary epithelial cells leads to mitochondrial dysfunction. (A)** Examination of the mitochondrial complex I activity in scrambled shRNA (NC) or NDUFS1 shRNA infected A549 cells. **(B)** Measurement of the abundance of total NAD, NADH, NAD+ and NAD+/NADH ratio in scrambled shRNA (NC) or NDUFS1 shRNA infected A549 cells. **(C)** Fluorescence imaging analysis of mitochondrial ROS in scrambled shRNA (NC) or NDUFS1 shRNA infected A549 cells. Scale bars: 250μm. Data were presented as mean ± SD. *p < 0.05, **p < 0.01, ***p < 0.001. Unless indicated, data were the representative of two independent experiments.

We wanted to investigate the mechanisms by which NDUFS1 modulated the expression of ENaCα. Research has demonstrated that mitochondrial dysfunction such as respiratory chain inhibition and increased ROS generation inhibited ENaC expression in collecting duct principal cells 26. Since NDUFS1 is essential for the activation of mitochondrial respiratory complex I that maintains mitochondrial functions 18. We speculated NDUFS1 may regulate ENaCα by mediating mitochondrial function. Firstly, we detected the activity of complex I in pulmonary epithelial cells. NDUFS1 deficiency largely reduced the activity of mitochondrial complex I in pulmonary epithelial cells (Fig.4A), which led to the dysfunction of the mitochondrial respiratory chain. Since the primary role of NDUFS1 in respiratory complex I is to regulate the generation of NAD+ 27, we next detected the abundance of total NAD, NADH, NAD+ and ratio of NAD+/NADH. Our data confirmed that the production of total NAD and NAD+ was significantly decreased while NADH was dramatically increased in NDUFS1 deficient pulmonary epithelial cells compared to control cells, leading to a great reduction of the ratio of NAD+/NADH (Fig.4B). Respiratory complex I dysfunction and NAD+ reduction led to increased ROS production 28, 29. We further examined the ROS expression levels in NDUFS1 deficient epithelial cells via DHE staining and found a significantly increase in total ROS levels in NDUFS1 deficiency epithelial cells (Fig.4C). Taken together, these results suggested that NDUSF1 deficiency in lung epithelial cells led to mitochondrial dysfunction such as reduced complex I activity, impaired NAD+ production and increased ROS, these may contribute to the decline of ENaCα.

Mitochondrial dysfunction is correlated with mitophagy and cell apoptosis, the two processes be well recognized to mediate the damage of pulmonary epithelial cells during ALI 30, 31. Our results confirmed the increased levels of PINK1 (a mitophagy marker) and cleaved caspase-3 (an apoptotic marker) in the PQ-induced lung injury model (Supplementary Fig.2A-B), underscoring the importance of both processes in mediating lung injury progression. Moreover, NDUFS1-deficient cells showed PINK1 upregulation but reduced cleaved caspase-3 levels, suggesting NDUFS1 is primarily essential for maintaining mitochondrial homeostasis but not sufficiently leading to cell death. Additional PQ stimulation further amplified PINK1 increases in NDUFS1-deficient cells, whereas NDUFS1 deficiency-mediated reduction of cleaved caspase-3 showed no significant change after PQ treatment (Supplementary Fig.2B). Collectively, these observations indicate that NDUFS1 deficiency primarily exacerbates mitochondrial damage in alveolar epithelial cells.

### NDUFS1 promotes ENaCα expression and alveolar fluid clearance in acute lung injury through mediating NAD+

**Figure 5:**
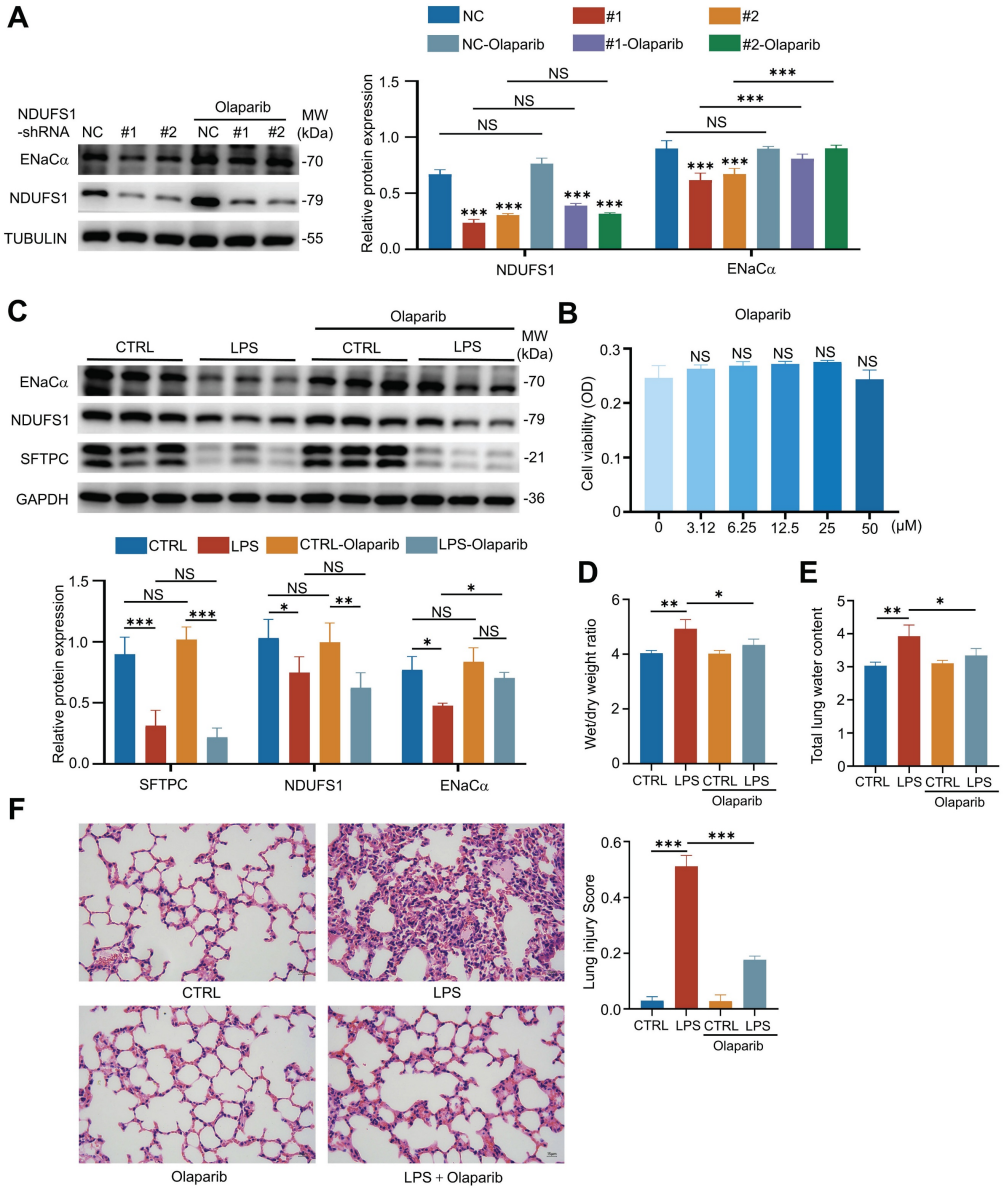
** NDUFS1 regulates ENaCα expression by modulating NAD+. (A)** Western blot analysis of NDUFS1 and ENaCα abundance in scrambled shRNA (NC) or NDUFS1 shRNA infected A549 cells with or without 50 µM Olaparib treatment for 24 h. **(B)** SRB analysis of cell viability with Olaparib treatment, as indicated, in shRNA (NC) affected A549 cells for 24 h. **(C)** Western blot analysis of NDUFS1 and ENaCα abundance in lung tissues from LPS-induced ALI mice model with or without Olaparib treatment. Mice were treated with LPS for 2h. n=3 mice per group. **(D)** Wet/dry weight ratio analysis in lung tissues from LPS-induced ALI mice model with or without Olaparib treatment. Mice were treated with LPS for 2h. n=3 mice per group. **(E)** Total lung water content in lung tissues from LPS-induced ALI mice model with or without Olaparib treatment. Mice were treated with LPS for 2h. n=3 mice per group. **(F)** Hematoxylin and eosin staining of lung tissues from LPS-induced ALI mice model with or without Olaparib treatment and the lung injury score of the tissues. Mice were treated with LPS for 2h. Scale bars: 15 µm. n=3 mice per group. Data were presented as mean ± SD. *p < 0.05, **p < 0.01, ***p < 0.001. NS, not statistically significant. Unless indicated, data were the representative of two independent experiments.

Literature has reported that NDUFS1 was mainly to regulate NAD generation in respiratory complex I 27. Next, we wondered whether NDUFS1 maintains ENaCα expression during ALI via NAD+ production. It has been reported that NAD+ could be utilized by three major routes 32, including PARP family members, SIRT family members and CD38. Several clinical available PARP inhibitors have been reported to be effective in treating cancer progression 33, including Olaparib, Niraparib and Rucaparib, which promote the accumulation of NAD+ in cells. We therefore examined the effect of PARP inhibitors on ENaCα expression in NDUFS1 deficient pulmonary epithelial cells. Unexpectedly, we found that only Olaparib treatment specifically normalized the expression of ENaCα in NDUFS1 deficient cells at the concentration not showing large impairment on cell viability (Fig.5A-B, Supplementary Fig.1A-B). We then treated LPS- or PQ-induced ALI mice model with Olaparib and found that the downregulated expression of ENaCα in mice lung tissues under either LPS or PQ stimulation was largely recovered (Fig.5C, Supplementary Fig.3A). Importantly, Olaparib treatment also relieved the symptom of pulmonary edema in LPS or PQ stimulated mice model, indicated by Wet/dry weight ratio and total lung water content (Fig.5D-E, Supplementary Fig.3B-C). Consistently, pathological analysis further revealed that LPS-induced prominent thickening of the alveolar wall, alveolar cavity collapse and alveolar flooding in lung tissues could be ameliorated with addition of Olaparib (Fig.5F). Taken together, these findings provided evidence that NDUFS1 regulated the ENaCα via NAD+ to promotes alveolar fluid clearance, and facilitated the recovery of ALI.

## Discussion

Acute lung injury (ALI) is caused by various pulmonary and extrapulmonary factors, including direct damage of intrinsic cells like epithelial cells, which leads to the accumulation of fluid in the alveoli and intractable hypoxemia 34, 35. Acute respiratory distress syndrome (ARDS) is a severe stage of ALI and claims millions of lives worldwide each year 36. Currently, the main treatment for ALI/ARDS is supportive therapies, including lung protective ventilation, prone positioning, and a fluid conservative strategy, together with anti-inflammation therapies. However, these therapies are not available to reduce the mortality rate 37, 38. An insufficient understanding of the pathological mechanism on ALI/ARDS limits the development of potential precise strategies for improving ALI/ARDS. In this study, we identified the importance of NDUFS1 in preventing the vicious cycle of acute lung injury via maintaining the expression of ENaCα for alveolar fluid clearance that improves the gas exchange function of pulmonary epithelial cells. Importantly, NDUFS1 is well-known to convert NADH into NAD+ while accumulation of NAD+ in pulmonary epithelial cells by treatment with Olaparib, the clinical available PARP inhibitor promotion of NAD+ accumulation, normalized the reduced expression of ENaCα and lung damage during ALI. Olaparib has also reported to ameliorate the inflammatory responses during ALI 39. Therefore, Olaparib would be a strategy of kill two birds with one stone, which is worth to examine to treat ARDS in clinic.

In physiological conditions, alveolar fluid clearance** (**AFC) relies on the synergistic transport of Na+ by epithelial sodium channel (ENaC) and Na-K-ATPase in alveolar epithelial cells 40. In the early stages of ALI, ENaC or Na-K-ATPase deficiency impairs alveolar fluid absorption 41. ENaC is the main driving force of AFC that facilitates the clear of excess fluid from alveoli. Mice lacking ENaCα were unable to remove edema fluid from alveolar spaces and died within 40 h of birth 42. Studies have shown that ENaCα is regulated by multiple factors, such as cholinergic, Urokinase-like plasminogen activator (uPA), steroid hormone, and Protein Kinase C 40. In this study, we further identified that either pharmacological or genetic inhibition of NDUFS1 significantly decreased the expression of ENaCα whereas NDUFS1 overexpression reversed the reduced ENaCα abundance during ALI. This indicated the potential role of NDUFS1 in enhancing alveolar fluid clearance in ALI. ENaC distributes widely in the epithelial cells of organs throughout the body and has been involved in the occurrence and development of many diseases 43. ENaC in epidermis promotes skin epidermal differentiation and ENaC deficiency in epithelial cells can inhibit wound healing 44, 45. ENaC dysfunction in hair cells of inner ears leads to disturbances in inner ear fluid balance and hearing loss 46. In addition, ENaC also participates the progression of diseases such as cystic fibrosis and diabetic kidney disease 47, 48. Our study first discovered that NDUFS1 played an important role in regulating ENaCα, which may provide a new idea for therapy of acute lung injury and other diseases.

Mitochondrial complex I is composed of 45 subunits and it is the beginning part of the respiratory chain 27. NDUFS1 subunit is the largest part of the functional N-module of mitochondrial complex I and is responsible for the oxidation of NADH to NAD+ 18, 49. Importantly, NDUFS1 plays a vital role in preserving the stability and function of the mitochondrial complex I, regulating the generation of ATP and ROS 50. A prior study showed that NDUFS1 knockdown in C. elegans resulted in neuronal and mitochondrial functional damage, reduced ATP generation, and increased ROS generation 51. In diabetic cardiomyopathy, AKAP1 deficiency in cardiomyocytes blocked the translocation of NDUFS1 from the cytosol to mitochondria, which reduced OXPHOS and raised the production of ROS 52. These observations raised the importance of NDUFS1-mediated mitochondrial activity in maintaining organ functions. Our study further revealed a reduced expression of NDUFS1 in mouse lung tissues and alveolar epithelial cells after exposure to LPS or PQ, two insults well-known to raise ALI. Of note, we detected downregulated NDUFS1 protein and mRNA levels in murine lung tissues 2 hours post-LPS intratracheal instillation. LPS instillation only minimally activates pulmonary apoptosis within 2 hours 53, making less possibility of cell death in directly reducing NDUSF1 expression. Furthermore, PQ stimulation reduced NDUFS1 abundance even in NDUFS1-overexpressing cells, and NDUFS1 deficiency correlated with PQ-induced mitochondrial damage, which NDUFS1 deficiency in alveolar epithelial cells led to a decrease in mitochondrial complex I activity, increased ROS generation, and impairment of ENaCα expression. Together, these results indicate that NDUFS1 reduction during lung injury is mainly attributable to mitochondrial damage. As NDUFS1 deficiency in lung tissues also resulted in decreased ENaCα expression, impaired AFC and alveolar edema, we speculated that NDUFS1-mediated mitochondrial homeostasis is important for the regulation of ENaC and clearance of alveolar fluid and the recovery of acute lung injury.

NAD (Nicotinamide Adenine Dinucleotide) was first discovered in 1906 and described as an ingredient that could improve the fermentation rate of yeast 54. NAD is the chemical backbone without charge, which is necessary for oxidation-reduction 55. NAD+ and NADH (Nicotinamide adenine dinucleotide hydrogen) refer to the oxidized and reduced forms of NAD, respectively. NAD+ was identified as a cofactor associated with energy metabolism and diverse metabolic pathways such as glucolipid metabolism and tricarboxylic acid cycle 56. NAD+ also plays a vital role in different physiological processes, including redox homeostasis, genomic stability, immunity and inflammation 57, 58. NAD+ synthesis includes three classical pathways: the Preiss-Handler pathway (PHP), the *de novo* synthesis pathway (DNP) and the salvage pathway (SP) 59. In addition, NAD+ can be cleaved by NAD+-consuming enzymes such as sirtuins, SARM1, polysaccharide polymerase (PARP) and cyclic ADP-ribose (cADPr) synthases CD38 and CD157 60. The abnormal metabolism of NAD+ thus leads to multiple diseases, including infection, apoptosis, tumorigenesis, age-associated neurodegeneration disorders, etc. In our research, increasing NAD+ via Olaparib treatment upregulated ENaCα protein expression in NDUFS1 deficient cells and improved alveolar fluid clearance (AFC) in LPS-induced ALI. We further identified the potential role of NAD+ for maintaining the expression of ENaCα and thus AFC to ameliorate ALI. Considering NDUFS1 mainly facilitates the production of NAD+ and NAD+ is widely required in different cell types, NAD+ would be the critical factor to maintain ENaCα expression in physiological conditions as well as in pathological conditions with no matter what insults are. Our study and other researches therefore provided the possibility that the homeostasis of ENaCα abundance would be largely dependent on NAD+ mediated cellular metabolism, including energy supplement and protein translational modification, which requires further efforts to elucidate. However, our work is the first to establish a direct link between NDUFS1, NAD+, and ENaC, providing a comprehensive framework for understanding how these components interact to maintain alveolar fluid clearance.

ALI involves complex multicellular interactions among alveolar epithelial cells, macrophages, fibroblasts, and other cell types. Our single-cell RNA sequencing mining analysis revealed that NDUFS1 expression is predominantly enriched in epithelial cells, justifying our focus on this population. NDUFS1 promotes alveolar fluid clearance specifically through ENaCα regulation in alveolar epithelial cells. Whether NDUFS1 regulates the activity of macrophages, fibroblasts, or other cell types by mediating ENaCα is yet to be elucidated. While our study did not directly examine other cell types, existing literature indicates mitochondria critically regulate macrophage polarization, immune responses, and fibroblast activation 61, 62. Given NDUFS1's essential role in mitochondrial complex I function and homeostasis 18, it would be reasonable to investigate the importance of the NDUFS1-mitochondrial homeostasis-ENaCα axis in other cell types during ALI in future studies.

## Conclusion

In conclusion, our research found that NDUFS1 was declined in alveolar epithelial cells during PQ/LPS-induced acute lung injury, which impaired ENaCα and alveolar fluid clearance. We further demonstrated that NDUFS1 maintained ENaCα expression and following alveolar fluid clearance via generation of NAD+, which provides a potential new theoretical basis for the treatment of acute lung injury.

## Supplementary Material

Supplementary figures and table.

## Abbreviations

PQ: paraquat
LPS: lipopolysaccharide
ALI: acute lung injury
ARDS: acute respiratory distress syndrome
AFC: alveolar fluid clearance
ENaC: epithelial sodium channel
NAD: nicotinamide adenine dinucleotide
NADH: nicotinamide adenine dinucleotide hydrogen
