# Supplementary material for: NDUFS1 upregulates ENaCα by NAD+ to promote alveolar fluid clearance in acute lung injury
Source: Int J Med Sci. 2025 Jul 28;22(13):3477–89. doi: 10.7150/ijms.112248 (PMC12375134; doi:10.7150/ijms.112248)
Supplement: Supplementary file 1 — Supplementary figures and table. [file ijmsv22p3477s1.pdf]

NDUFS1 upregulates ENaCα by NAD+ to promote alveolar fluid clearance in acute lung injury

Mengmeng Wang[1#], Mengting Chen[1#], Jianping Zhu[1], Yu Zhang[1], Jian Lu[3], Zhiying Yue[2], Zhengfeng Yang[1,2*], Ruilan Wang[1*]

[1] Department of Critical Care Medicine, Shanghai General Hospital, Shanghai Jiao Tong University School of Medicine, Shanghai 201620, China

[2] Precision Research Center for Refractory Diseases, Shanghai Jiao Tong University Pioneer Research Institute for Molecular and Cell Therapies, Shanghai General Hospital, Shanghai Jiao Tong University School of Medicine, Shanghai, 201620, China; State Key Laboratory of Innovative Immunotherapy, School of Pharmaceutical Sciences, Shanghai Jiao Tong University, Shanghai, 200240, China

[3] Department of Critical Care Medicine, Shanghai United Family Hospital, Shanghai, 200335, China

# These authors contributed equally to this work.

[*] To whom correspondence should be addressed:

Ruilan Wang, M.D.

Department of Critical Care Medicine, Shanghai General Hospital

Shanghai 201620, China

Email: wangruilang@sjtu.edu.cn

Zhengfeng Yang, Ph.D.

Precision Research Center for Refractory Diseases, Shanghai General Hospital

Shanghai 201620, China

Email: zhengfeng.yang@shgh.cn

Supplementary Table 1.

	Gene		Sequence
Homo	Ndufs1	sense	5’ TTAGCAAATCACCCATTGGACTG 3’
		antisense	5’ CCCCTCTAAAAATCGGCTCCTA 3’
Homo	Ndufs2	sense	5’ GTCCGATTGCCGATTCAGC 3’
		antisense	5’ GCTTGGGTACATAACAGCTCC 3’
Homo	Ndufs3	sense	5’ ACTGTCAGACCACGGAATGAT 3’
		antisense	5’ GGGCAAGATTTCAGCCACATAC 3’
Homo	Ndufs7	sense	5’ CTTCGCAAGGTCTACGACCAG 3’
		antisense	5’ GGAATAGTGGTAGTAGCCTCCTC 3’
Homo	Ndufs8	sense	5’ CCATCAACTACCCGTTCGAGA 3’
		antisense	5’ CCGCAGTAGATGCACTTGG 3’
Homo	Nudfv1	sense	5’ AGGATGAAGACCGGATTTTCAC 3’
		antisense	5’ CAGTCACCTCGACTCAGGGA 3’
Homo	Ndufv2	sense	5’ GGTTGGGGAGACTACACCTGA 3’
		antisense	5’ CTTGGCCCTGGTTTTGGGAT 3’
Homo	ENaCα	sense	5’ TCTGCACCTTTGGCATGATGT 3’
		antisense	5’ GAAGACGAGCTTGTCCGAGT 3’
Homo	β-actin	sense	5’ CATGTACGTTGCTATCCAGGC 3’
		antisense	5’ CTCCTTAATGTCACGCACGAT 3’
Mouse	Ndufs1	sense	5’ AGGATATGTTCGCACAACTGG 3’
		antisense	5’ TCATGGTAACAGAATCGAGGGA 3’
Mouse	ENaCα	sense	5’ CCTTCTCCTTGGATAGCCTGG 3’
		antisense	5’ CAGACGGCCATCTTGAGTAGC 3’
Mouse	β-actin	sense	5’ GTGACGTTGACATCCGTAAAGA 3’
		antisense	5’ GCCGGACTCATCGTACTCC 3’

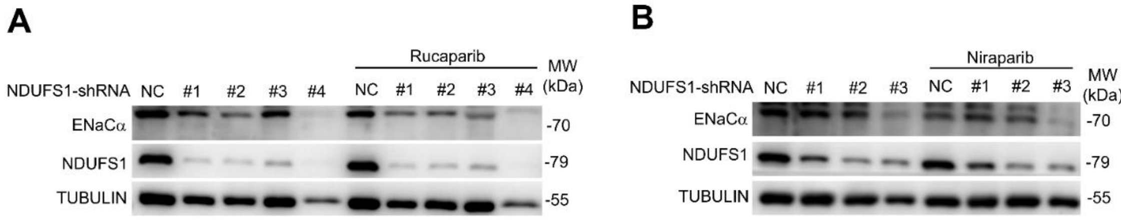

Supplementary Figure 1. The effect of Niraparib and Rucaparib on ENaCα expression in NDUFS1 deficient pulmonary epithelial cells. (A) Western blot analysis of NDUFS1 and ENaCα abundance in scrambled shRNA (NC) or NDUFS1 shRNA infected A549 cells with or without Rucaparib treatment for 24 h. **(B)** Western blot analysis of NDUFS1 and ENaCα abundance in scrambled shRNA (NC) or NDUFS1 shRNA infected A549 cells with or without Niraparib treatment for 24 h.

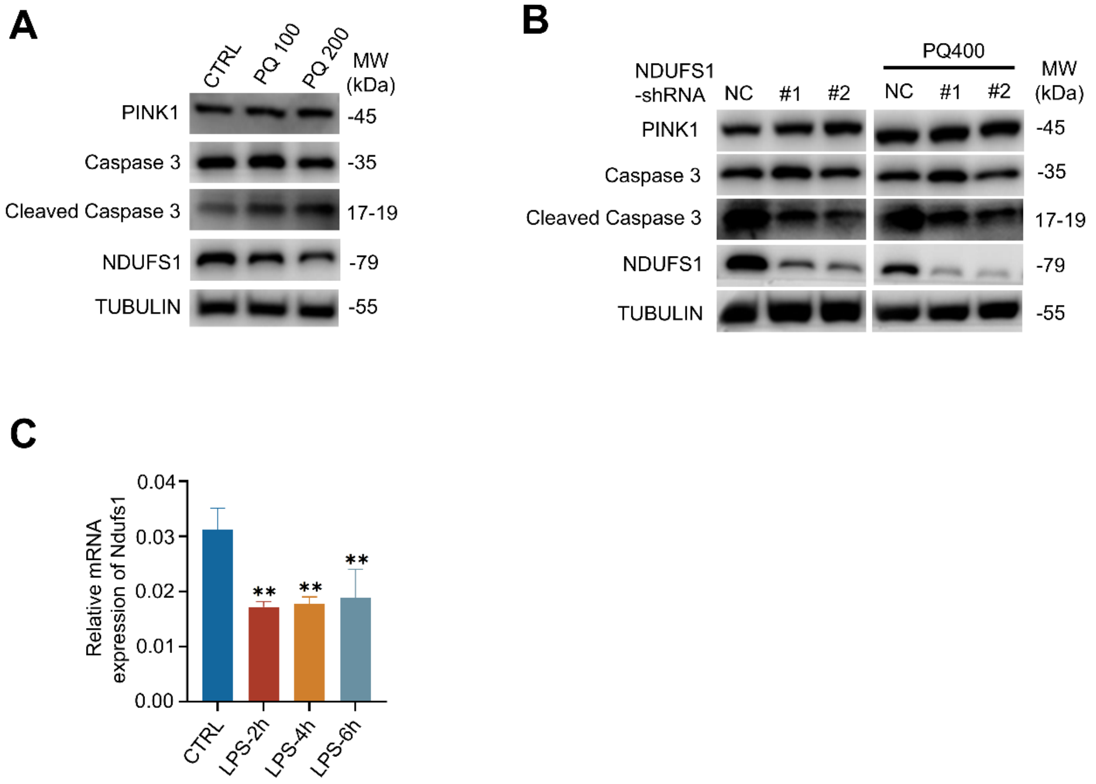

Supplementary Figure 2. The level of cell death and mitophagy marker in PQ-induced ALI models and NDUFS1 knockdown A549 cell line. (A) Western blot analysis of PINK1, Caspase3, Cleaved Caspase3 and NDUFS1 abundance, as indicated, in A549 cells treated with or without 100 or 200 μM PQ for 12 h. **(B)** Western blot analysis of PINK1, Caspase3, Cleaved Caspase3 and NDUFS1 abundance in scrambled shRNA (NC) or NDUFS1 shRNA infected A549 cells with or without 400 μM PQ for 12h. **(C)** QPCR analysis of NDUFS1 abundance in lung tissues from LPS-induced ALI mice model. Mice were treated with LPS for 2h, 4h and 6h. Data were presented as mean ± SD. $*p < 0.05$, $**p < 0.01$. Unless indicated, data were the representative of two independent experiments.

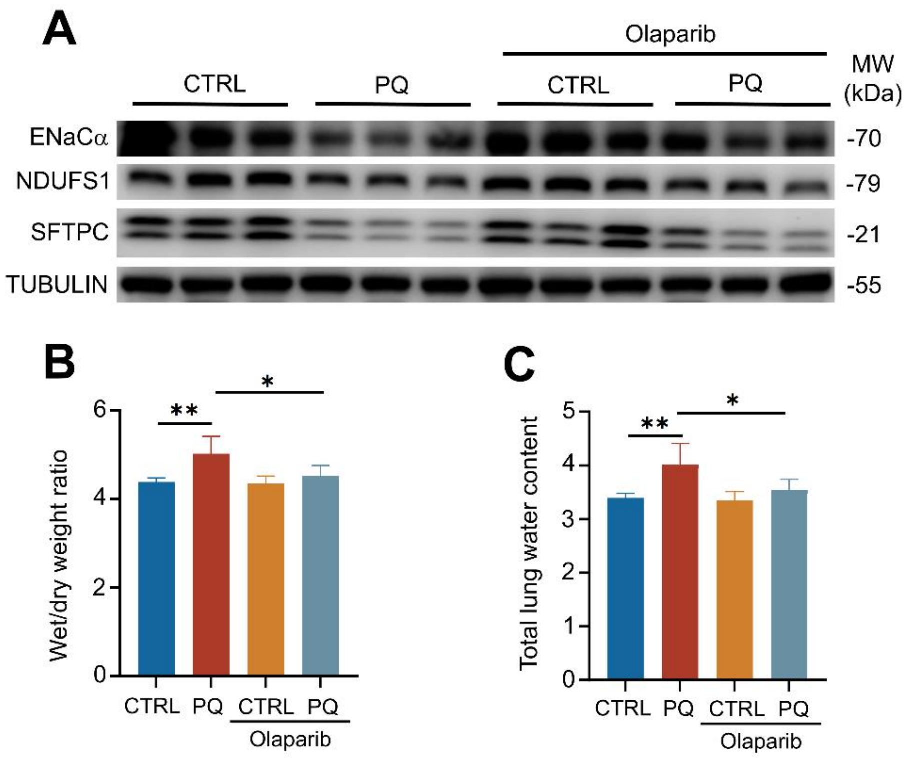

Supplementary Figure 3. NDUFS1 regulates ENaCα expression by modulating NAD+ in PQ-induced acute lung injury. (A) Western blot analysis of NDUFS1 and ENaCα abundance in lung tissues from PQ-induced ALI mice model with or without Olaparib treatment. Mice were treated with PQ for 6h. **(B)** Wet/dry weight ratio analysis in lung tissues from PQ-induced ALI mice model with or without Olaparib treatment. Mice were treated with PQ for 6h. **(C)** Total lung water content in lung tissues from PQ-induced ALI mice model with or without Olaparib treatment. Mice were treated with PQ for 6h. Data were presented as mean ± SD. $*p < 0.05$, $**p < 0.01$. Unless indicated, data were the representative of two independent experiments.
